# Interspinous Process (ISP) Devices in Comparison to the Use of Traditional Posterior Spinal Instrumentation

**DOI:** 10.7759/cureus.13886

**Published:** 2021-03-14

**Authors:** Jordan E Faulkner, Kareem Khalifeh, Junko Hara, Burak Ozgur

**Affiliations:** 1 Neurological Surgery, Hoag Memorial Hospital, Newport Beach, USA; 2 Neurosurgery, ONE Brain and Spine Center, Irvine, USA; 3 Neurosurgery, Pickup Family Neurosciences Institute, Newport Beach, USA; 4 Neurosurgery, Hoag Memorial Hospital, Newport Beach, USA

**Keywords:** spinal fusion, isp, pedicle screw, instrumentation, spine surgery

## Abstract

A systematic literature review was conducted on studies comparing interspinous process (ISP) devices to traditional methods of posterior spinal instrumentation (pedicle screw-rod construct), in terms of indications of use, complications, pain assessment, estimated blood loss, length of hospital stay, reoperation rates, and return to work. The objective was to analyze, evaluate and summarize the current published literature on the proposed efficacy and clinical and surgical long-term outcomes of the ISP device in comparison to the traditional posterior spinal instrumentation (pedicle screw-rod construct). The ISP device is a minimally invasive and less disruptive alternative to traditional methods of posterior spinal instrumentation (pedicle screw-rod construct). However, very few published literature studies to date have reported the comparison of ISPs in terms of efficacy and clinical and surgical outcomes, to traditional posterior spinal instrumentation.

A systematic literature review was performed in PubMed and Google Scholar to evaluate the results of published research that meet the defined inclusion and exclusion criteria and to analyze clinical indications and surgical outcomes of the ISP device compared to traditional methods of posterior spinal instrumentation (pedicle screw-rod construct). Inclusion criteria included keywords such as “ISP device, ISP, posterior spinal instrumentation, pedicle screw fixation, bilateral pedicle screws, interbody fusion with posterior spinal instrumentation, lumbar spinal stenosis, and posterior lumbar stability.” No exclusion criteria keywords were included in this literature review.

ISPs provide a high degree of spinal stability in multiple planes, including a decreased range of motion restriction in flexion-extension, and comparable results to bilateral pedicle screw (BPS) in axial rotation. The use of the ISP device in adjunct with an interbody fusion, ensures less estimated operative blood loss (EBL), shorter operative time, less bony exposure without the need for extensive soft tissue or muscle retraction, a decrease in the rate of pseudoarthrosis, and a shorter length of hospital stay (LOHS) when compared to the traditional posterior instrumentation (pedicle screw-rod construct).

Based on the various published literature reviews noted throughout this research paper, it is safe to conclude, that an ISP device that is accompanied by interbody fusion, including posterior approaches posterior lumbar interbody fusion (PLIF) and transforaminal lumbar interbody fusion (TLIF); anterior approaches such as anterior interbody fusion (ALIF), and lateral approaches including direct lateral interbody fusion (DLIF), lateral lumbar interbody fusion (LLIF), extreme lateral interbody fusion (XLIF), is considered a credible and an effective minimally invasive option for the treatment of mild to moderate lumbar stenosis and stable low-grade spondylolisthesis (less than two) when compared to the traditional posterior spinal instrumentation of a pedicle screw-rod construct. Surgeons that are relatively new to the ISP technologies for spinal instrumentation would likely benefit from more clinical and surgical evidence of safety and efficacy in published peer-reviewed medical literature. Further clinical trials are needed to manifest the efficacy of ISPs regarding postoperative outcomes when compared to traditional posterior instrumentation techniques (pedicle screw-rod construct) with adjunct interbody fusions.

## Introduction and background

Posterior spinal instrumentation is frequently used as the main surgical treatment for spinal instability and pain related to underlying spinal pathologies. Spinal instability can result from numerous sources including congenital defects, trauma or injury, degenerative disc disease, adult degenerative scoliosis, spondylolisthesis, neoplastic diseases of the spine, and stenosis. The main objective of posterior spinal instrumentation is to provide spinal stability, by encouraging adjacent vertebrae to fuse. The posterior spinal instrumentation may eventually break and fatigue without adjunct interbody fusion {including posterior approaches such as posterior lumbar interbody fusion (PLIF) and transforaminal lumbar interbody fusion (TLIF); anterior approaches such as anterior interbody fusion (ALIF); and lateral approaches such as direct lateral interbody fusion (DLIF), lateral lumbar interbody fusion (LLIF) and extreme lateral interbody fusion (XLIF)}. Spinal instrumentation and minimally invasive spinal surgical techniques have evolved over the years to achieve successful spinal stabilization after spinal decompression. The current, most widely used method of posterior spinal instrumentation is the bilateral pedicle screw-rod construct, used in adjunct to interbody fusion. However, over the previous decades, the minimally invasive interspinous process (ISP) device has earned interest and maybe potentially utilized further in the future, more so than that of the traditional method (pedicle screw-rod construct), for the surgical treatment of spinal pain and stability to achieve improved patient satisfaction. The use of the ISP device in adjunct with an interbody fusion, ensures less estimated operative blood loss (EBL), shorter operative time, less bony exposure without the need for extensive soft tissue or muscle retraction, a decrease in the rate of pseudoarthrosis, and a shorter length of hospital stay (LOHS) when compared to the traditional posterior instrumentation (pedicle screw-rod construct) [1]. Indications for use of posterior spinal instrumentation (pedicle screw -rod construct) include but are not limited to: unstable vertebral fractures, instability caused by mild to moderate spinal stenosis, degenerative adult scoliosis, post-laminectomy spondylolisthesis, painful pseudoarthrosis, stabilization of spinal osteotomies, and augmenting anterior strut grafting for tumors and/or infections. Two methods of pedicle-screw rod constructs are currently used for posterior spinal instrumentation, the bilateral pedicle screw (BPS) fixation, and the unilateral pedicle screw (UPS) fixation techniques. In general, BPS fixation in adjunct to an interbody fusion is preferred as a standard procedure due to its rigid fixation, great biomechanical stability, and good clinical results [2, 3]. UPS is also commonly used in adjunct to interbody fusion and has multiple advantages in reduced soft tissue disruption of the contralateral side, shorter surgical time, and lower implant costs [4-6], and provides relatively less rotational stability and stiffness [7]. Although the UPS technique may have reduced operative time and blood loss, it represented a lower fusion rate than the BPS construct [8]. Both the BPS and UPS fixation techniques are technically demanding and associated with high complication rates, including but not limited to deep wound infections from extensive dissection, increased rate of cerebrospinal fluid leakage, and neurological complications [9]. Jutte et al. retrospectively analyzed 105 pedicle screw fixation operations and found complications of varying severity in 54% of the patients. Such complications include but are not limited to deep wound infections found in 4.7% of the patients, screw misplacement found in 6.5% of the patients, and screw breakage which occurred in 12.4% of the patients [9]. The pedicle screw-rod construct (BPS and UPS) was once known as one of the only spinal surgical options to treat spinal instability and provide spinal stabilization, however, given extensive operative times, significant operative blood loss, increased length of hospitalization stay, and extensive tissue and muscle dissection noted in the pedicle screw rod construct (BPS and UPS), viable alternatives such as the ISP devices have been developed and are gaining acceptance among surgeons and patients.

ISP devices were originally designed to function as standalone, non-fusion devices; however, newer, evolving designs have been used in conjunction with an inter-body fusion and/or lumbar decompression, as an alternate method of posterior spinal fixation [1, 10, 11]. The first ISP device was developed in the 1950s, however, due to its design flaws, material, surgical technique, and applied indications, its use was discontinued [12]. In 1986, the first modern ISP device, the Wallis system, also known as a “floating system”, was composed of a titanium spacer placed between the spinous processes and secured with two Dacron ligaments wrapped around the spinous processes [12]. A second generation of the Wallis device was designed with a slightly different shape and was composed of polyetheretherketone (PEEK) material [12]. Fashioned out of silicone into the shape of a dumbbell to off-load the facet joints and decrease the intradiscal pressure, the Minns device was the first “soft” interspinous spacer indicated for sagittal plane instability [12]. In the 1990s, several other ISP devices displaying significant differences in design, materials, surgical techniques, and indications appeared in Europe and South America [12]. Kaech et al. first reported on the interspinous “U” (Coflex) suggesting that it was indicated for protection against adjacent level disc disease and restabilization of a lumbar laminectomy. Caserta et al. reported on the device for intervertebral assisted motion (DIAM) implant, which was indicated for a number of conditions, including degenerative disc disease, herniated nucleus pulposus, and lumbar instability. In 2005, the X-Stop device (Medtronic, Tolochenaz, Switzerland) was approved by the US Food and Drug Administration (USFDA) for the treatment of neurogenic intermittent claudication secondary to lumbar stenosis [12]. October of 2012, the USFDA approved the coflex® Interlaminar Stabilization® device for the treatment of lumbar spinal stenosis [12]. Various ISP devices have been recently introduced to the lumbar spine market as an alternative to traditional pedicle-screw fixation surgery in managing symptomatic lumbar spinal pathology, especially in the older population. Despite the fact that ISPs are composed of a wide range of different materials including, but not limited to titanium, Ti-Bond® technology, polyetheretherketone, and elastomeric compounds; the aim of ISP devices is to unload the lumbar spine, restore foraminal height, and stabilize the lumbar spine by distracting the spinous processes [11].

The ISP device has been proposed to be used as a stand-alone device following a lumbar decompression, in patients who suffer from mild to moderate lumbar spinal stenosis. Classified as a minimally invasive spinal surgery, the ISP spares muscle, tissue, and ligaments, encourages fusion, and minimizes complications that allow for quicker post-operative recovery time, compared to the traditional pedicle screw-rod construct, for the treatment of degenerative disc disease, low-grade spondylolisthesis, and lumbar spinal stenosis and/or lumbar spinal instability. ISPs are contraindicated in pars fractures and high-grade spondylolisthesis. The purpose of an ISP device is to restore foraminal height, maintain proper alignment, distract the spinous process, and provide stability for the degenerating spine [12]. It is important to note here that there are now numerous versions of ISP devices on the market. Some are designed as fusion devices while some are not. In this article, we report our experience using a device as a fusion and instrumentation construct.

## Review

The purpose of the current retrospective literature review was to compare reported data points for such clinically important outcomes such as estimated blood loss, length of hospital stay, improvements in pain via the visual analog scale (VAS) measure, and the reduction in dysfunction via the Oswestry disability index (ODI).

A literature search was performed in PubMed and Google Scholar for English publications including keywords such as “ISP device, ISP, posterior spinal instrumentation, pedicle screw fixation, PLIF, TLIF, ALIF, DLIF, LLIF, XLIF, bilateral pedicle screw fixation, unilateral pedicle screw fixation, interbody fusion with posterior spinal instrumentation, and posterior lumbar stability.” No exclusion criteria keywords were included in this literature review. All findings were summarized qualitatively without statistical pooling or performing a meta-analysis.

Results

Spinal Stability in Relation to Pain 

Lumbar spinal instability can be defined as a loss of a normal pattern of spinal motion, that is commonly known to cause low back pain and/or neurologic dysfunction. Achieving lumbar spinal stability depends on the posterior spinal instrumentation fixation device and the current and long-term conditions of the lumbar spine, in regards to the density and quality of bone. The ability of the posterior spinal structures, specifically the spinous processes, have adequate structural integrity to accommodate stress forces exerted by an ISP [13]. Shepherd et al. measured the mechanical force required to fracture the spinous process of 32 specimens with average to below-average bone mineral density [14]. A significant linear correlation between bone mineral density and bone strength was established; a mean load of 339 N was required to cause a spinous process failure with 95% confidence interval of 257 to 447 N [14]. In another study, Talwar et al. tested the incidence of spinous process fracture during ISP implantation, which demonstrated a lateral load of 95 to 786 N that was required to cause failure of the posterior spinal elements with no significant difference in load tolerance between the cranial, middle or caudal aspects of the spinous process, whereas an insertional load of 11 to 150 N was required for ISP fixation [15]. This wide variance in insertion load was thought to be primarily based on bone density but could not be definitively correlated. However, based on these results, an intraoperative lumbar spinous process fracture is less likely to occur in osteopenia patients with an ISP implantation, although an intraoperative lumbar spinous process fracture remains very probable for patients with osteoporosis. ISP devices have demonstrated a degree of biomechanical spinal stability, the ability to maintain structural spinal integrity, accommodate mechanical stress forces, and preserve adjacent level structures [13]. ISP devices are placed at the level of lumbar stenosis and positioned between two adjacent spinous processes. The distractive force applied by the ISP device and the subsequent height restoration is believed to be the main mechanism through which it functions, reducing movement of the lumbar vertebrae and decompressing the impinged spinal nerves [16]. ISP devices provide lumbar spinal stability in lumbar flexion and lumbar extension, equivalent to traditional BPS fixation, and provides limitation of axial rotation and lateral bending motion, equal to that of BPS fixation [10]. The pedicle screw-rod construct (BPS and UPS) fixation was designed with the intention to reduce spinal instability. Pedicle screws are not only used to reduce spinal instability but are being procedurally used to surgically treat various spinal modalities, such as degenerative scoliosis, kyphosis, vertebral resection, and osteotomies [17]. Pedicle screws are inserted within the pedicle cortices of the vertebrae and act as anchors for connection of rods while crosslinks adjoining the traversing rods are also an option. Pedicle screws capture the strongest part of the lumbar vertebrae, enhancing the bone-screw interface and permitting the application of higher corrective forces. Interbody fusion, specifically, PLIF with pedicle screw fixation has been linked to adjacent segmental degeneration (ASD) due to the additional forces on the facet joints at adjacent levels [10, 18, 19]. ASD can be defined as degeneration that develops at mobile segments above or below a fused spinal segment and usually develops after an interbody fusion or other lumbar spinal procedures. A study of 76 adult patients by Kim et al. showed significantly more adjacent level angular hypermobility and degenerative changes in the pedicle screw stabilization group [20]. Regarding range of motion, Wang et al. studied the biomechanical characteristics of an ISP on 109 cadaveric specimens in an in vitro test and determined that the ISP achieved excellent mean restriction of range of motion (ROM) [10]. Flexion-extension ROM decreased significantly to 4.14° for ISP with lumbar interbody fusion when compared to the intact spine (10.1°) whereas flexion-extension ROM for pedicle screw fixation (BPS) with lumbar interbody fusion decreased to 5.03° when compared to the intact spine (10.1°) [10]. ISPs have been recognized to preserve and maintain normal spinal anatomy without disruption of adjacent facet joints, which results in less hardware-related pain or accelerate adjacent force degeneration when compared to BPS fixation [1]. Clinical studies indicate that the application of any posterior spinal fixation (BPS and UPS) to the painful segment of the lumbar spine can significantly reduce low back pain [21].

Complications of Pedicle Screw-Rod Fixation

The most common complications associated with the use of traditional pedicle screw-rod constructs (BPS and UPS) include increased rates of cerebrospinal fluid (CSF) leakage, neural, vascular, or dural injury, deep wound infection, significant radiation exposure to the people in the operating room during implantation, and hardware/mechanical failure [9,22]. Jutte et al. further identified and studied the technical demands and high complication rates in 105 consecutive operations associated with the pedicle-screw-rod fixation technique [9]. Jutte found complications varying in severity in 54% of the patients, with deep wound infections in 4.7% of the patient, screw misplacement in 6.5% of the patients and screw breakage in 12.4% of the patients [9]. Jutte found that although majority of the resulted complications were able to be managed by reoperation and treatment with wound debridement and antibiotics, he also recommended a combination of the pedicle screw-rod fixation in adjunct to and interbody fusion.

Complications of Interspinous Process (ISP) Devices

ISPs are considered a minimally invasive surgical alternative to that of BPS, however, ISPs still present with various complications. Gazzeri et al. further studied one thousand one hundred eight patients, for a minimum of three years after ISP placement, who were affected by symptomatic one- or two-level segmental lumbar spine degenerative disease [23]. The complication rate was 7.8%. There were 27 fractures of the spinous process and 23 dura mater tears with CSF leakage. The reported dura mater tears with CSF leakage were likely due to operative or postoperative complications, such as surgical techniques, poor tissue characteristics such as a surgical revision, scar tissue and/or trauma, and not the ISP device itself. The ultimate failure rate requiring additional surgery was 9.6%. The reasons for revision, which always involved removal of the original implant, were acute worsening of low-back pain or lack of improvement (45 cases), recurrence of symptoms after an initial good outcome (42 cases), and implant dislocation (20 cases) [23]. Gazzeri concluded that in the same study, ISP should not be used as a substitute for pedicle-screw rod fixation (BPS) in cases of major spinal instability and severe spondylolisthesis. He also reported that over distraction, poor bone density (osteoporosis), and poor patient selection may all be factors in the development of complications [23].

Estimated Blood Loss (EBL)/Operative Risk

The ISP implantation has been proven to result in less EBL, less risk of infection, shorter operative time, less bony exposure without the need for extensive soft tissue or muscle retraction, a decrease in the rate of pseudarthrosis, and a shorter LOHS/recovery [1]. A study on 32 patients (21 ISP and 11 BPS) from Wang et al. found that the ISP plate is not only easy to implant but is also associated with minimal operative risk [1]. Kim et al. reported in a study of 76 adult patients a significantly shorter operation time and 50% lower EBL in patients stabilized with interspinous fixation as supplementation to a posterior approach for interbody fusion compared to those that were stabilized using transpedicular fixation/traditional posterior spinal instrumentation such as the pedicle screw-rod construct (BPS) [20]. Panchel et al. reported from their study, that the ISP patients mean posterior intraoperative metrics were: blood loss, 71mL; mean operating time 52 minutes; incision length 5.5 cm; and fluoroscopic imaging time, 10 seconds [24]. The BPS patients mean posterior intraoperative metrics were: blood loss, 120 mL; mean operating time 79 minutes, incision length, 7.2cm; and fluoroscopic imaging time, 57.4 seconds [24]. In another study, Wang et al. reported in ISP treated patients, the median operative EBL (75 ml) was lower than in BPS treated patients {open BPS (150 ml); tubular BPS (125 ml)} [1]. In another study comparing the UPS fixation with the BPS fixation, Liu et al. reported the UPS had a shorter operative time and less EBL than the BPS group (P<0.01) [8]. In regards to the geriatric population, minimal blood loss and less bony exposure without the need for extensive soft tissue or muscle retraction, reduces postoperative complications (such as anemia, prolonged pain, reduced risk of infection, and exacerbation of co-morbidities), which leads to a decrease in postoperative discomfort and significant improvement in VAS scores in the ISP patients and BPS patients [20].

Pain Assessment

Tatsumi et al. reported on postoperative results for 55 patients with an ISP in adjunct with an interbody fusion, who demonstrated clinical and statistically significant improvement in ODI scores at six weeks and three months. Panchel et al. reported the mean improvements in ODI scores for patients postoperatively from a baseline of six weeks, to three months, to six months, to 12 months [24]. The mean improvements in ODI scores for patients with an ISP were respectively, 13, 22, 24, and 26 points. Statistically significant ODI score improvement was achieved in ISP patients by six weeks postoperatively (P <0.01) and further maintained out to 12 months (P <0.01) [24]. Similarly, six-week, three-month, six-month, and 12-month score improvements for posterior screw fixation (PSF) patients were 9, 20, 19, and 22, respectively (P < 0.01) [24]. Non-inferiority (10-point mean difference) of ODI score improvement, relative to baseline, at 12 months was demonstrated by the ISP group compared with the PSF control group (mean difference, 3.60 points; 95% confidence interval [CI], 7 3.62 to 10.81 points) [24].

Length of Hospital Stay

LOHS postoperatively, following posterior spinal instrumentation, depends on a plethora of patient variables including but not limited to age, weight/BMI (body mass index), co-morbidities, and the specific type of posterior spinal instrumentation device. LOHS has been further studied and analyzed to examine possible ways to reduce the cost associated with posterior spinal instrumentation procedures. In one study, Wang et al. reported the median LOHS was three days for both the IPD and tubular/percutaneous BPS fixation group, but four days in the open BPS fixation group [1]. El-Kadi et al. reported patients who underwent UPS and/or BPS by one surgeon at one facility, had positive correlations with LOHS, indicating that those with higher age and BMI would be expected to have a longer length of stay [25]. Segura-Trepichio et al. reported LOHS was significantly lower in the discectomy plus ISP when compared to the PLIF group with microdiscectomy, with an average LOHS of two days versus an average LOHS of five days(P < 0.01) [26]. Patients undergoing discectomy plus ISP will have a decrease in the mean LOHS when compared to that obtained with discectomy plus PLIF [26].

Reoperation Rates

Although there are more reported reoperative rates in BPS groups, there is also a significant amount of published literature pertaining to BPS reoperative rates vs. ISP reoperative rates. Pintauro et al. analyzed and compared the first generation ISP with the next-generation ISP in terms of complications based on thirty-seven studies included from 2011 to 2016 [16]. He reported ISP failure occurred at a mean of 3.7%, with a lower tendency to happen with next-generation ISPs. Reoperations occurred at a lower rate with the next-generation ISP, with a mean follow-up of 24 months (3.7% vs. 11.1%). The clinical outcome was not influenced by the type/brand of ISP [16]. Gazzeri et al. conducted a study that indeed was influenced by the type of ISP (Coflex) and compared Coflex implantation with decompression and PLIF w/ BPS [12]. The percentage of adverse events was 5.6% for both groups with a reoperation rate of 10.7% in the ISP (Coflex) group and 7.5% in the PLIF w/ BPS group [12]. Wang et al. reported in his study of 32 patients with rigid ISP SPIRE fixation, that there were no instances of major surgery-induced complications, pseudoarthrosis, or hardware failure during a mean follow-up period of 5.5 months [1]. Best et al. reported sixty-seven patients have had at least a two-year follow-up. Twenty-four patients had a posterior fusion with pedicle screws, and 43 had translaminar facet screw fixation [27]. Nine patients of the pedicle screw population (37.5%) had a reoperation to remove their instrumentation. Two patients of the translaminar facet screw population (4.7%) had reoperations on their lumbar spine. There was a significant association between posterior instrumentation type and reoperation (P = 0.001) [27].

Return to Work

Given the apparent minimally invasive benefits of ISP, this has shown to lead to less postoperative discomfort and immediately improved postoperative VAS scores in the ISP patients, and although the BPS patients also showed significant improvement postoperatively, they required a longer rehabilitation period [21]. Regarding ISP return to work, future analysis of larger cohort studies with longer follow-ups with additional published literature is still needed to further assess clinical outcomes.

Limitations

Limitations were encountered when researching complications of ISPs, as only one specific ISP device (X-STOP) was accounted for and numerous research on complications of the ISP was greater than two decades. Regarding ISP return to work, future analysis of larger cohort studies with longer follow-ups with additional published literature is still needed to further assess patient satisfaction. ISPs cannot be used in patients with high-grade spondylolisthesis and in patients with a pars fracture. The use of multilevel ISP’s may be challenging, depending on the specific ISP device used.

## Conclusions

An ISP device that is accompanied by interbody fusion, including posterior approaches PLIF and TLIF, anterior approaches ALIF, and lateral approaches DLIF, LLIF, XLIF, is considered an effective minimally invasive option for the treatment of mild to moderate lumbar stenosis and stable low-grade spondylolisthesis when compared to the traditional posterior spinal instrumentation of a pedicle screw-rod construct (BPS and/or UPS). Other minimally invasive techniques like microscopic and tubular interbody fusions (percutaneous TLIF for example) have already shown efficacy and improved data in decreased operating room (OR) time, lower infection rates, lower cost, less use of postoperative narcotics, and shorter LOHS when compared to traditional techniques. ISPs continue to remain early in the published literature due to insufficient clinical and surgical evidence of safety and efficacy in published peer-reviewed medical literature. Further clinical trials are needed to further manifest the efficacy of ISPs (stand-alone vs. in adjunct to lumbar interbody fusion) regarding postoperative outcomes when compared to traditional posterior instrumentation techniques (pedicle screw-rod construct) with adjunct interbody fusions. Given the significant evolution of ISPs devices over the years, clinical and surgical outcomes will vary depending on the type of ISP device used. Despite the specific type of ISP used, the aim of ISP devices is to unload the lumbar spine, restore foraminal height, and stabilize the lumbar spine by distracting the spinous processes. It is important to note that there are indeed fusion and non-fusion ISP devices on the market. It is up to the surgeon which they intend to use as indicated. Additional studies should be performed and reported in the future commenting on the fusion rates as well as the patient functional outcomes. Anatomic and biomechanical studies should also be performed as this technology evolves and is used more often. Certainly, specific conditions as osteoporosis and osteopenia will have an effect on any instrumentation construct. 
